# Early surgery vs conservative management among asymptomatic aortic stenosis: A systematic review and meta-analysis

**DOI:** 10.1016/j.ijcha.2022.101125

**Published:** 2022-09-22

**Authors:** Vikash Jaiswal, Nida Khan, Akash Jaiswal, Mehak Dagar, Amey Joshi, Helen Huang, Hira Naz, Abdelrahman M. Attia, Mohammed Ghanim, Abiram Baburaj, David Song

**Affiliations:** a Department of Cardiovascular Research, Larkin Community Hospital, FL, USA; b Jinnah Sindh Medical University, Karachi, Pakistan; c Department of Geriatric Medicine, All India Institute of Medical Science, New Delhi, India; d Himalayan Institute of Medical Science, Dehradun, India; e University of Medicine and Health Science, Royal College of Surgeons in Ireland, Dublin, Ireland; f Fatima Jinnah Medical University, Pakistan; g Faculty of Medicine, Cairo University, Cairo, Egypt; h Henry Ford Healthcare System, Detroit, MI, USA; i Center for Public Health, Queen’s University Belfast, UK; j Department of Internal Medicine, Icahn School of Medicine at Mount Sinai – Elmhurst Hospital Center, NY, USA

**Keywords:** Asymptomatic Aortic Stenosis, Conservative management, Outcomes, Mortality, ACM, All-cause mortality, SCD, Sudden Cardiac Death, HF, Heart failure, MI, Myocardial infarction, NA, Not available, OR, Odds ratio, RCT, Randomized controlled trial, PSM, Propensity score match

## Abstract

**Introduction:**

Although aortic valve replacement in severe symptomatic Aortic Stenosis (AS) are clearly outlined, the role of surgical intervention in asymptomatic severe AS remains unclear with limited evidence. The aim of our meta-analysis is to evaluate the efficacy and safety of early surgical aortic valve repair compared to conservative management.

**Methods:**

A systematic literature search was performed in PubMed, Scopus, Embase and Cochrane databases for studies comparing the early surgery versus conservative management among asymptomatic aortic stenosis patients. Unadjusted odds ratios (OR) were pooled using a random-effect model, and a p-value of < 0.05 was considered statistically significant.

**Results:**

A total of 5 articles (3 observational studies and 2 randomized controlled trials) were included. At a median followup of 4.1 years, here were significantly lower odds of all-cause mortality [OR = 0.30 (95 %CI:0.17–0.53), p < 0.0001], cardiovascular mortality [OR = 0.35 (95 %CI:(0.17–0.72), p = 0.005], and sudden cardiac death (OR = 0.36 (95 %CI: 0.15–0.89), p = 0.03) among early surgery group compared with conservative care. There was no significant difference between incidence of major bleeding, clinical thromboembolic events, hospitalization due to heart failure, stroke and myocardial infarction between the conservative care groups and early surgery.

**Conclusion:**

Among asymptomatic patients with AS, early surgery shows better outcomes in reducing all-cause mortality and cardiovascular mortality compared with conservative management approaches.

## Highlights

• Aortic valve replacement remains the definitive choice of treatment for AS.
• All-cause mortality and adverse events are lower with early surgical valve repair.
• In high-risk AS patients, early surgery is safer than conservative management.
• Early intervention is crucial to prevent irreversible damage of AS.

## 1 Introduction

Aortic stenosis (AS) is a cause of significant health burden in developed countries affecting nearly 5% of the population with increasing prevalence with increasing age [1]. Due to the lack of pharmacological treatment for the prevention or treatment of AS, aortic valve replacement (AVR) has been the definitive choice of therapy [2]. Although the indications for AVR in severe symptomatic AS are clearly outlined [3], [4], the role of surgical intervention in asymptomatic severe AS remains unclear with limited evidence [2].

Conventional guidelines recommend a watchful waiting strategy in asymptomatic severe AS with prompt surgical intervention at the onset of symptoms [5]. This recommendation was based on the premise that the potential mortality benefit of surgical intervention may not outweigh its operative risk [3], [6], [7]. However, this was primarily predicated on small cohorts and single-center observational studies. Recent alarming literature on the high prevalence of asymptomatic severe AS (37–46%) [8], with nearly half progressing to symptomatic status requiring AVR, has questioned the timing of the surgical intervention in this population [9]. Furthermore, a deeper understanding of the potentially irreversible impact of AS on the myocardium even after AVR has raised interest in early intervention strategies [10].

Currently, the 2020 American Heart Association/American College of Cardiology (AHA/ACC) guidelines recommend AVR in asymptomatic severe AS in selected cases (class Ib, IIa, IIb recommendation) which excludes those with preserved left ventricular ejection fraction (LVEF) [4]. We conducted this meta-analysis in light of the recent data from the RECOVERY trial (Randomized Comparison of Early Surgery versus Conventional Treatment in Very Severe Aortic Stenosis) and the AVATAR trial (Aortic Valve Replacement Versus Conservative Treatment in Asymptomatic Severe Aortic Stenosis) which suggested marked reduction in a composite of all-cause mortality in severe asymptomatic AS and preserved LVEF with early AVR [11], [12].

## 2 Methods

This study was carried out in accordance with the Preferred Reporting Items for Systematic Review and Meta-analysis (PRISMA) 2020 checklist and was performed according to established methods, as described previously [13], [14], [15].

### 2.1 Outcomes

The primary outcome of interest for this meta-analysis was all-cause mortality. The secondary outcomes of interest were the incidence of cardiovascular mortality (CVM), sudden cardiac death (SCD), hospitalization due to HF, Clinical thromboembolic events, major bleeding, myocardial infarction (MI), and stroke.

### 2.2 Search strategy

We conducted a systematic literature search across the following databases: PubMed, Embase, Cochrane Library, and Scopus. Predefined Mesh terms were used, by applying the BOOLEAN (“AND” and “OR”) logic. The following search terms were used: “” The search was performed from inception until April 30, 2022, without any language or date restrictions. All the studies were carefully screened and exported to Endnote 2020 library (Clarivate Analytics, USA). Two reviewers (VJ and BA) reviewed the studies based on title and abstract. A third author (AJ) arbitrated discrepancies regarding the inclusion of studies.

### 2.3 Eligibility criteria

#### 2.3.1 Inclusion criteria

1. Studies with patients aged ≥ 18 years.
2. Studies including intervention and control groups where the intervention group employed patients with early surgery, while the placebo/control group comprised patients with conservative management.
3. Studies were required to report at least one of the desired outcomes, i.e., all-cause mortality, risk of CVD, SCD, HF, major blending, MI and Stroke.
4. Eligible study designs included RCTs, prospective, retrospective, and propensity score matched studies.

#### 2.3.2 Exclusion criteria

1. Animal studies, abstracts, editorials, commentaries, systematic reviews, single patient case studies, letters, and studies with insufficient data were excluded.
2. Studies where a single arm was presented without comparators, and with non-compliant outcomes were also excluded.

### 2.4 Data extraction and quality assessment and statistical analysis

Data of the eligible selected studies such as demographic, comorbidities, risk factors, and outcomes of both groups were extracted into a shared spreadsheet by two authors (VJ and AB).

Two investigators (VJ and AI) independently appraised the potential risk of bias using Cochrane Collaboration’s tool for assessing risk of bias in randomized controlled trials and the Newcastle-Ottawa (NOS) scale for observational studies [16], [17]. We then classified studies low, moderate, or high quality based on the scores after evaluation (**S. Table 2, S. Table3)**

Baseline continuous variables were summarized as mean (SD), whereas dichotomous variables were described as frequencies or percentages. A conventional, two-arm meta-analysis for primary and secondary outcome was performed. We used the Review Manager (RevMan) Version 5.4 (Nordic Cochrane Center, The Cochrane Collaboration, 2012, Copenhagen, Denmark) software to calculate the pooled effect size with odds ratio (OR) and 95% confidence interval (CI) by the Mantel‐Haenszel method for dichotomous outcomes [18]. For continuous variables, the mean difference for both groups were compared to determine the net effect size by the Inverse Variance method. The results are presented graphically in forest plots. Both fixed- and random-effect models were used. The random-effect model was used when there was significant heterogeneity across studies. I^2^ statistics evaluated the heterogeneity of studies. The probability value of p < 0.05 was considered statistically significant, and the pooled estimates were reported with a 95% confidence interval (CI). According to the recommendations, we converted the median and interquartile ranges into mean and standard deviation according to the recommendations [19].

## 3 Result

### 3.1 Study selection

The preliminary database search using the pre-specified keywords yielded 2075 articles, of which 974 studies were excluded after removal of duplicates. 1026 studies were further excluded post initial title and abstract screening based on the inclusion and exclusion criteria and comparison arm (ES and CM groups). Full text reviews of 75 studies was considered to be eligible for further evaluation. 70 studies were further excluded as they either had unmatching target populations, were not primary research articles, letters, review, commentary, symptomatic patients data, outcomes of interest not given, or lacked a comparison arm. Hence, a total of 5 studies that met the eligibility criteria were included in our meta-analysis in which 2 studies are randomized clinical trials [11], [12], and 3 studies are prospective in nature [20], [21], [22] (Table 1**)**. The Preferred Reporting Items for Systematic Reviews and Meta-Analyses (PRISMA) flow diagram is depicted in **Supplementary** Fig. 1.

**Table 1:** Baseline characteristic of included studies arranged in early surgery vs conservative management form.

**Variables**	**AVATAR Trail, 2021**[11]	**RECOVERY Trail, 2021**[12]	**Taniguchi et al, 2015** [20]	**Kim et al, 2019** [21]	**Kang et al, 2010** [22]
Study Design	RCT	RCT	PSM	Prospective	Prospective
Sample Size	78/79	73/72	291/291	221/247	102/95
Age, Mean (SD)	68/69.12	65/63.4	71.6/77.8	61/67.1	63/63
Male, %	59/55.7	37/34	43.30/42.6	50/51	54/46
Follow up, Years	2.3/2.1	6.2/6.1	3.7/3.7	5.1/5.1	4.1/4.1
Aortic Stenosis Type	Severe	Very Severe	Severe	Severe	Very Severe
Inclusion Criteria	Asymptomatic patients. Severe AS (AVA < 1 cm^2^, Vmax > 4 m/s or MG > 40 mm Hg). Negative exercise tolerance test.	Asymptomatic patients. Very severe AS (AVA < 0.75 cm^2^, Vmax > 4.5 m/s or MG > 50 mm Hg). Exercise testing was selectively performed to evaluate patients with non-specific symptoms	Asymptomatic Patients. AVA < 1.0 cm^2^; Vmax > 4.0 m/s; MAG > 40 mmHg	Asymptomatic patients. AVA ≤ 1.0 cm^2^; Vmax ≥ 4.0 m/s; MAG ≥ 40 mmHg	Asymptomatic patients .AVA ≤ 0.75 cm^2^ plus either Vmax ≥ 4.5 m/s or MAG ≥ 50 mmHg
DM, n	14/23	13/7	59/66	37/66	10/10
HTN, n	69/70	40/39	188/187	92/122	37/39
Dyslipidemia, n	31/28	41/42	116/83	–	–
CAD, n	1/3	5/1	61/74	32/17	–
PVD, n	0/1	1/2	23/31	2/4	–
Previous Stroke, n	2/2	3/3	–	9/34	–
Atrial Fibrillation, n	–	3/6	39/40	19/34	–
Bicuspid Aortic Valve, n	–	49/39	–	126/63	57/39
Degenerative valvular disease, n	–	22/26	–	–	33/45
Rheumatic Heart Disease, n	–	2/7	–	25/42	12/11
LV mass index g/m^2^, (Mean)	160.95/148.37	135.6/133.7	–	–	158/159
LVEF%	68.25/67.75	64.8/64.8	66.8/68.2	63.7/63.1	62/63
AVA, cm^2^ (Mean)	0.69/0.72	0.63/0.64	0.67/0.75	0.74/0.80	0.61/0.62

### 3.2 Baseline characteristics of included studies

A total of 5 studies were included in our analysis which included 765 patients in the early surgery group and 784 patients in the conservative care group [11], [12], [20], [21], [22]. The mean age for patients who underwent early surgery and conservative care was 65.72 years and 68.08 years respectively. 48.5% vs 47.45% accounted for the male population in the early surgery and conservative care group respectively. The most common comorbidity was hypertension (55.7% vs 58.3%), diabetes mellitus (17.4% vs 22%), coronary artery disease (15% vs 13.8%), rheumatic heart disease (9.84% vs 14.5%), atrial fibrillation (10.42% vs 13.11%), peripheral vascular disease (4% vs 5.5%), in both early surgery and conservative care groups respectively. The history of previous stroke amongst the early surgery and conservative care group was 3.76% vs 9.8% respectively. 58.6% vs 34.05% patient population were found to have bicuspid aortic valves in the early surgery and conservative care group respectively. 31.43% of the study population in the early surgery group had degenerative valvular disease as compared to 42.51% patients in the conservative care group. The mean aortic valve area (cm^2^) was (0.676 vs 0.71) and the mean LV mass index (g/m^2^) was (148.53 vs 150.9) in the early surgery and conservative care group respectively. The median follow-up duration was 4.1 years.

### 3.3 Risk of bias assessment

All the three observational studies were having low risk of bias, while the randomized clinical trials also showed low risk of bias on the quality assessment of the two included RCTs (**Supplementary Table 2**–**3**)

### 3.4 Meta analysis of clinical outcomes among included studies

#### 3.4.1 All-cause mortality

**Fig. 1:**
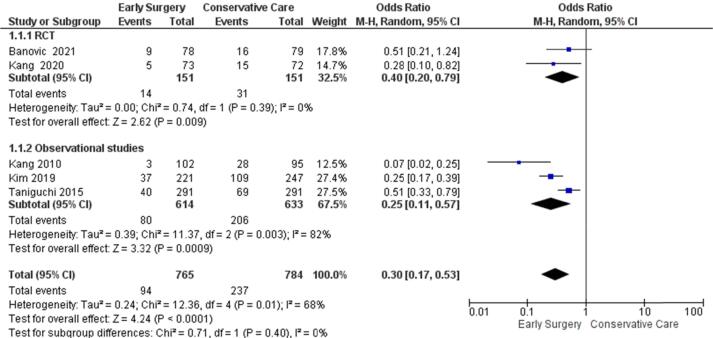
Primary outcome forest plot- random effect of all-cause mortality.

At a median follow-up of 4.1 years the odds of all-cause mortality in the Early Surgery group are significantly lower [OR = 0.30, (95 %CI:0.17–0.53), p < 0.0001, I^2^ = 68%] compared with conservative management groups. The test for subgroup differences indicates that there is no statistically significant subgroup effect (p = 0.40), though there is heterogeneity between studies reporting data on all-cause mortality for the observational studies subgroup (I^2^ = 82%) (Fig. 1).

#### 3.4.2 Cardiovascular mortality

**Fig. 2:**
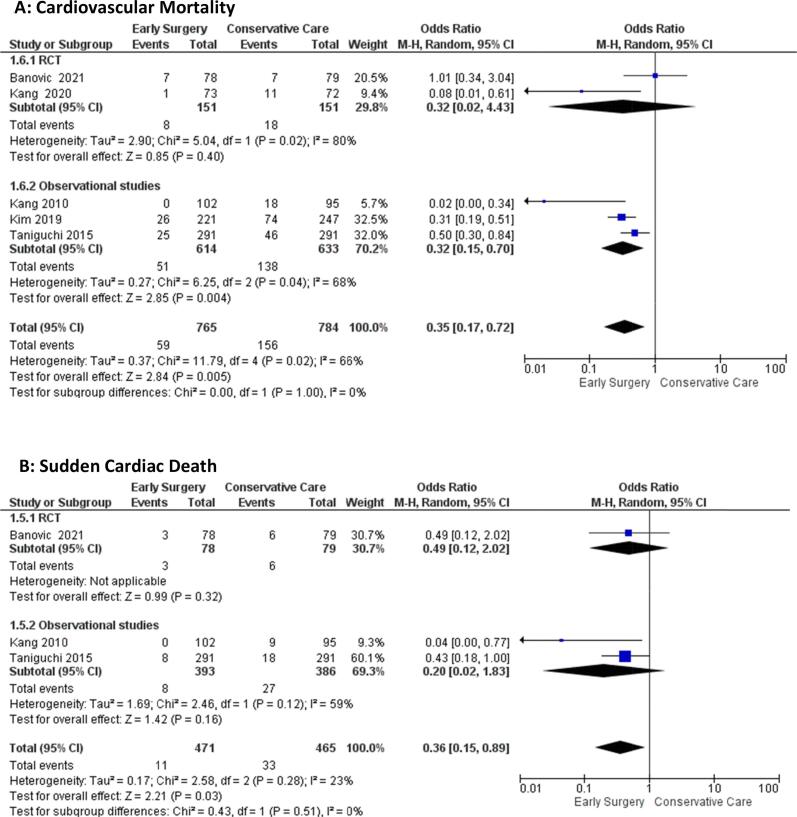
Forest plot of A) Cardiovascular Mortality, and B) Sudden Cardiac Death.

The odds of cardiovascular mortality in the Early Surgery group are significantly lower [OR = 0.35(95 %CI:(0.17–0.72), p = 0.005, I^2^ = 66%] than conservative management groups. The test for subgroup differences suggests that there is no statistically significant subgroup effect (p = 1.00). Heterogeneity among RCT’s measuring cardiovascular mortality is confirmed by high (I^2^ = 80%), while moderate heterogeneity among observational studies (I^2^ = 66%) (Fig. 2**A**).

#### 3.4.3 Sudden cardiac death

The odds of sudden cardiac death are significantly lower in the early surgery groups compared with conservative management groups [OR = 0.36(95 %CI: 0.15–0.89), p = 0.03, I^2^ = 23%]. The test for subgroup differences suggests that there is no statistically significant subgroup effect (p = 0.51) (Fig. 2**B**).

#### 3.4.4 Hospitalization for heart failure

**Fig. 3:**
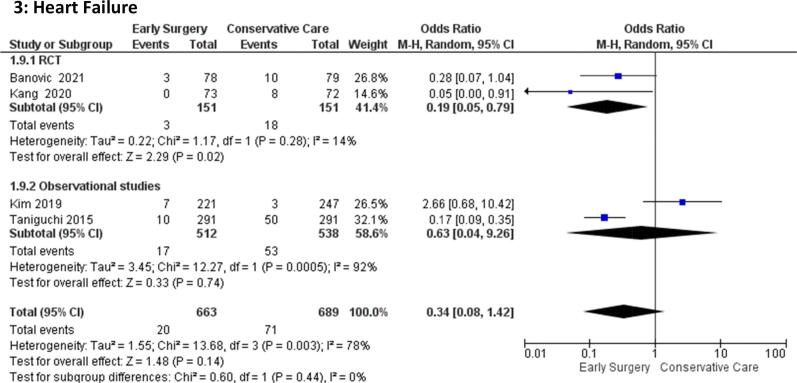
Forest plot of Heart Failure.

The odds of hospitalization due to sudden heart failure were comparable among early surgery groups [OR = 0.34(95 %CI:0.08–1.42), p = 0.14] and conservative management groups. The test for subgroup differences suggests that there is no statistically significant subgroup effect (p = 0.44). There is significant heterogeneity among the observational studies (I = 92%) **(**Fig. 3**)**

#### 3.4.5 Clinical thromboembolic events, major bleeding, myocardial infarction, stroke

**Fig. 4:**
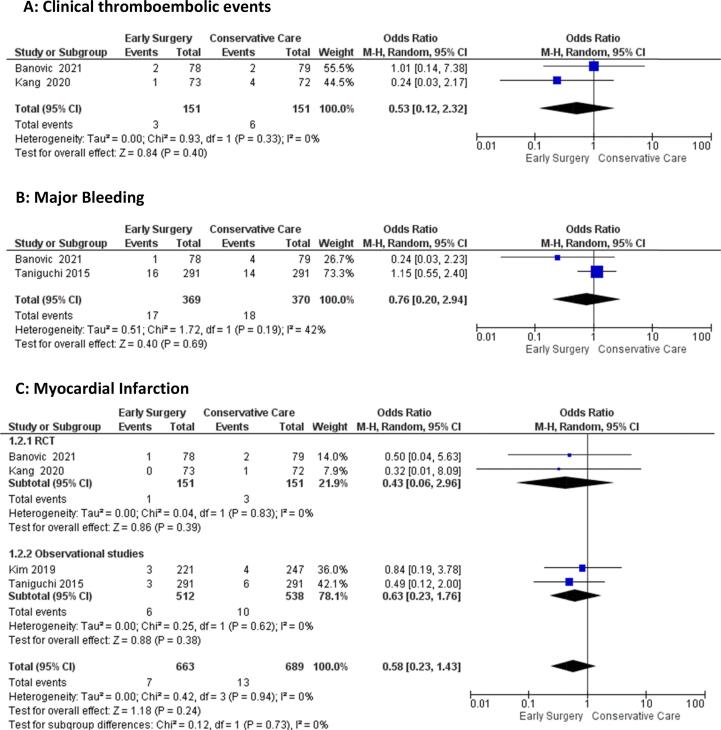

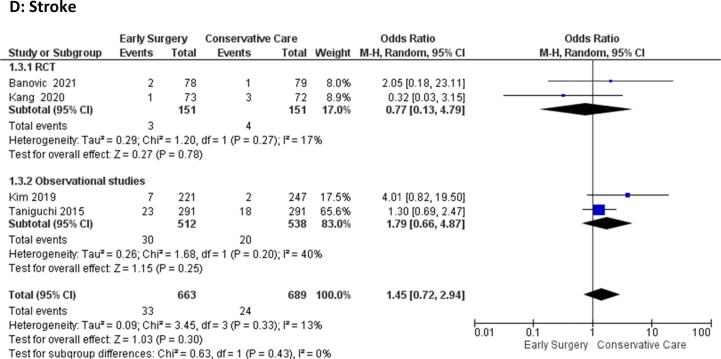
Forest plot of A) Clinical thromboembolic events, B) Major Bleeding, C) Myocardial Infarction, D) Stroke.

The odds of clinical thromboembolic events [OR = 0.53 (95 %CI:0.12–2.32), p = 0.40, I^2^ = 0%], major bleeding [OR = 0.76 (95 %CI:0.20–2.94), p = 0.69, I^2^ = 42%], Myocardial Infarction [OR = 0.58 (95 %CI:0.23–1.43), p = 0.24, I^2^ = 0%], and stroke [OR = 1.45 (95 %CI: 0.72–2.94), p = 0.30, I^2^ = 13%)] were comparable between the early surgery and conservative management groups **(**Fig. 4**A-D)**

## 4 Discussion

**Fig. 5:**
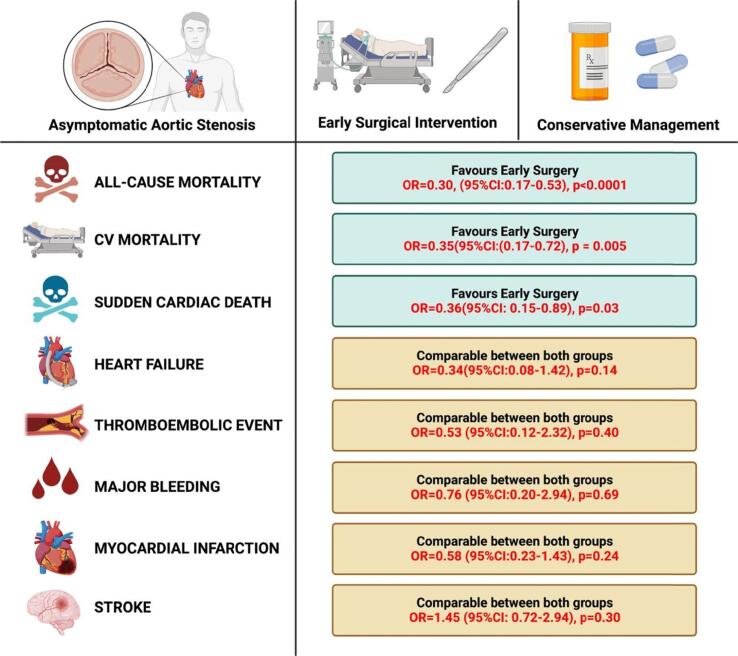
Central illustration for primary and secondary outcomes among early surgery vs conservative groups. (Original image created with biorender.com)

In this meta-analysis of 3 observational studies and 2 randomized control trials - RECOVERY and AVATAR trial, patients with early surgery in asymptomatic severe aortic stenosis had lower all-cause mortality as compared to those with conservative care. We found no difference between both interventions regarding the complications including clinical thromboembolic events, major bleeding, myocardial infarction, hospitalization for heart failure and stroke. There were lower odds of cardiovascular mortality and sudden cardiac death in patients undergoing early surgical aortic valve replacement as summarized in Fig. 5**.**

Previous studies investigating the outcomes of early surgery and conservative management had encouraging findings. A meta-analysis by Yokoyama et al. demonstrates low all-cause mortality and cardiovascular mortality for early surgery groups and supported our findings. [23] This was also the case in a meta-analysis by Tsampasian et al. which analyzed the randomized control trials (RCTs) and observational studies separately. [24] While RCTs meta-analysis showed reduction in all-cause mortality after early surgery, there was also a reduction hospitalization for heart failure. There was no difference observed in the risk of cardiovascular death. Additionally, meta-analysis of observational studies showed improved mortality in patients given early intervention. [24] However, a meta-analysis by Ismayl et al. demonstrated that while a reduction in all-cause mortality was seen for early SAVR compared to watchful waiting, CVM and SCD were comparable between our groups. In comparison, our results showed a significant reduction in CVM and SCD among ES groups. [25] A lower risk of hospitalization for heart failure for early SAVR was also reported in the aforementioned study, an outcome that was comparable in our study amongst both groups. Previous studies mostly focused on limited outcomes in the early surgery group as compared to this meta-analysis of RCTs and observational studies, which focused on various outcomes together with the complications [23], [24], [25].

The potential benefit of early surgical intervention emerged as a safe option in high-risk patients with asymptomatic aortic stenosis (AS) as compared to conservative management. Patients with severe AS with left ventricular ejection fraction (LVEF) less than 60% at baseline and greater than 5 m/s peak aortic jet velocity have increased risks of mortality [2]. According to Lancellotti et al. the patients with these baselines were associated with greater all-cause and cardiovascular mortality without surgical aortic valve replacement [2]. Their 30 day post-procedure mortality was 0.9%, emphasizing the importance of early surgery [2]. The early aortic valve replacement (AVR), being the safe option in younger patients with small aortic valve area,high mean gradient on echocardiogram and higher Vmax, have lower 2 year (7.5% vs 16.1%) and 3 year (9.0% vs 21.1%) mortality rates as compared to watchful waiting [26]. The low mortality rates in AVR are due to higher ejection fraction and higher peak velocity [26].

Apart from the low all-cause mortality our meta-analysis also showed lower odds of cardiovascular mortality in the early surgery group. Pellikka et al. also depicted in his study that most asymptomatic patients with significant AS will not only develop symptoms within 5 years but have only 25% probability of escaping cardiac mortality as compared to patients receiving AVR [27]. The possibility of irreversible myocardial damage in asymptomatic patients can lead to systolic and diastolic dysfunction [28]. This accompanied by interstitial fibrosis and pulmonary hypertension as a result of valvular heart disease contribute to increased cardiovascular mortality and morbidity and risk of developing major adverse cardiovascular event in case the surgery is delayed [29], [30].

Our findings are in line with the recent meta-analysis conducted by Kumar et al. our study showed no significant difference in the odds of incidence of major bleeding, clinical thromboembolic events, stroke, and myocardial infarction between early surgery and conservative management [31]. The study by Kvaslerud et al. shows that 49% asymptomatic patients had increased MACE incidence by delaying surgery within 3 years [8]. The major contributing factors were N-terminal pro B-type natriuretic peptide (NT-proBNP) and history of coronary artery disease [8].

Although the sudden cardiac death in asymptomatic patients with severe AS are usually higher than in the general population, however in our meta-analysis the odds were significantly lower in the population receiving early aortic valve surgery [27]. There was no significant difference in the odds of incidence of hospitalization due to heart failure in this meta-analysis between patients receiving early surgery and conservative management as justified in another meta-analysis by Kumar et al. [31] The patient demographics play a vital role in heart failure hospitalization as according to Chen et al. The numerous factors are associated with heart failure hospitalization such as older age, lower body mass index and NYHA class IV together with prior myocardial infarction, renal insufficiency, percutaneous coronary intervention and pulmonary hypertension. It is emphasized that to maximize the clinical benefits, patients with severe AS should be treated earlier to avoid the risk of hospitalization.[32]

Numerous factors such as sustained pressure overload during watchful waiting together with functional and structural impairment of left ventricle and reduced left ventricular ejection fraction contribute to various adverse clinical effects, including the sudden cardiac death [29], [30].

## 5 Strength and limitations

This meta-analysis provides strong evidence that asymptomatic patients with severe AS may benefit from early surgical aortic valve replacement (SAVR) as compared to conservative management. The inclusion of 2 recent RCT; RECOVERY and AVATAR together with observational studies increased the power of analysis and strengthened the results. While our findings are clinically significant, there are certain limitations that merit consideration.

Most of the included studies were observational and nonrandomized, despite using several adjustment models, the residual selection bias and the possibility of ascertainment bias for symptoms cannot be excluded due to non-mandated exercise test patient inclusion. Therefore, the survival in the AVR was impacted by higher rates of morbidities. As some studies were conducted over an expanded time period, with patients having bicuspid aortic valves, impacting the study cohort, decision criteria and treatment strategies. This together with the patient follow-up in the multicenter study being less close as compared to single-center studies resulted in the underestimation of the emerging symptoms in AS. On the other hand, many studies have mentioned their outcomes in hazard ratio which were not pooled instead of raw numbers.

Despite the limitations, the evidence supporting early surgery in asymptomatic AS patients is compelling based on our results and previously reported meta-analyses. However, these findings warrant large-scaled investigation to support early surgical intervention into clinical practice due to the small and limited numbers of randomized control trials and small patient populations in existing studies. Ideally, future studies should focus on recruiting a larger cohort of patients with asymptomatic aortic stenosis to validate and emphasize our results in support of early surgical intervention over conservative management.

## 6 Conclusion

Among asymptomatic patients with AS, SAVR shows better outcomes in reducing mortality and other complications compared with conservative management. With the inclusion of two recent RCTs, the beneficial effect of early surgery approach may be considered among the asymptomatic patients by physicians and healthcare providers. Future RCTs with larger patient cohorts will help improve these results and solidify effective interventions for asymptomatic for aortic stenosis.

## 7 Data availability statement

All data used for the purpose of this study is available online or in the Supplementary file.

## 8 Source of funding

This research did not receive any specific grant from funding agencies in the public, commercial, or not-for-profit sectors. This research did not receive any specific grant from funding agencies in the public, commercial, or not-for-profit sectors.

### CRediT authorship contribution statement

**Vikash Jaiswal:** Conceptualization, Methodology, Writing – original draft, Writing – review & editing. **Nida Khan:** Writing – original draft. **Akash Jaiswal:** Writing – original draft. **Mehak Dagar:** Writing – original draft. **Amey Joshi:** Writing – original draft. **Helen Huang:** Writing – original draft, Writing – review & editing. **Hira Naz:** . **Abdelrahman M. Attia:** Formal analysis, Investigation. **Mohammed Ghanim:** Writing – original draft. **Abiram Baburaj:** Writing – original draft. **David Song:** Writing – review & editing.

## Declaration of Competing Interest

The authors declare that they have no known competing financial interests or personal relationships that could have appeared to influence the work reported in this paper.
