# Supplementary material for: Early surgery vs conservative management among asymptomatic aortic stenosis: A systematic review and meta-analysis
Source: Int J Cardiol Heart Vasc. 2022 Sep 22;43:101125. doi: 10.1016/j.ijcha.2022.101125 (PMC9513172; doi:10.1016/j.ijcha.2022.101125)
Supplement: Supplementary data 1 [file mmc1.docx]

**Supplementary legend**

**Supplementary Image 1**: PRISMA Flow of the search strategy for systematic review and meta-analysis.
**Supplementary Table 1:** Search strategy

**Supplementary Table 2:** Newcastle-Ottawa scale for quality assessment and bias assessment of observational studies

**Supplementary Figure 1:** Quality assessment using Cochrane Risk of Bias tool for randomized controlled trials (RCTs)

**Supplementary Image 1**: PRISMA Flow of the search strategy for systematic review and meta-analysis.

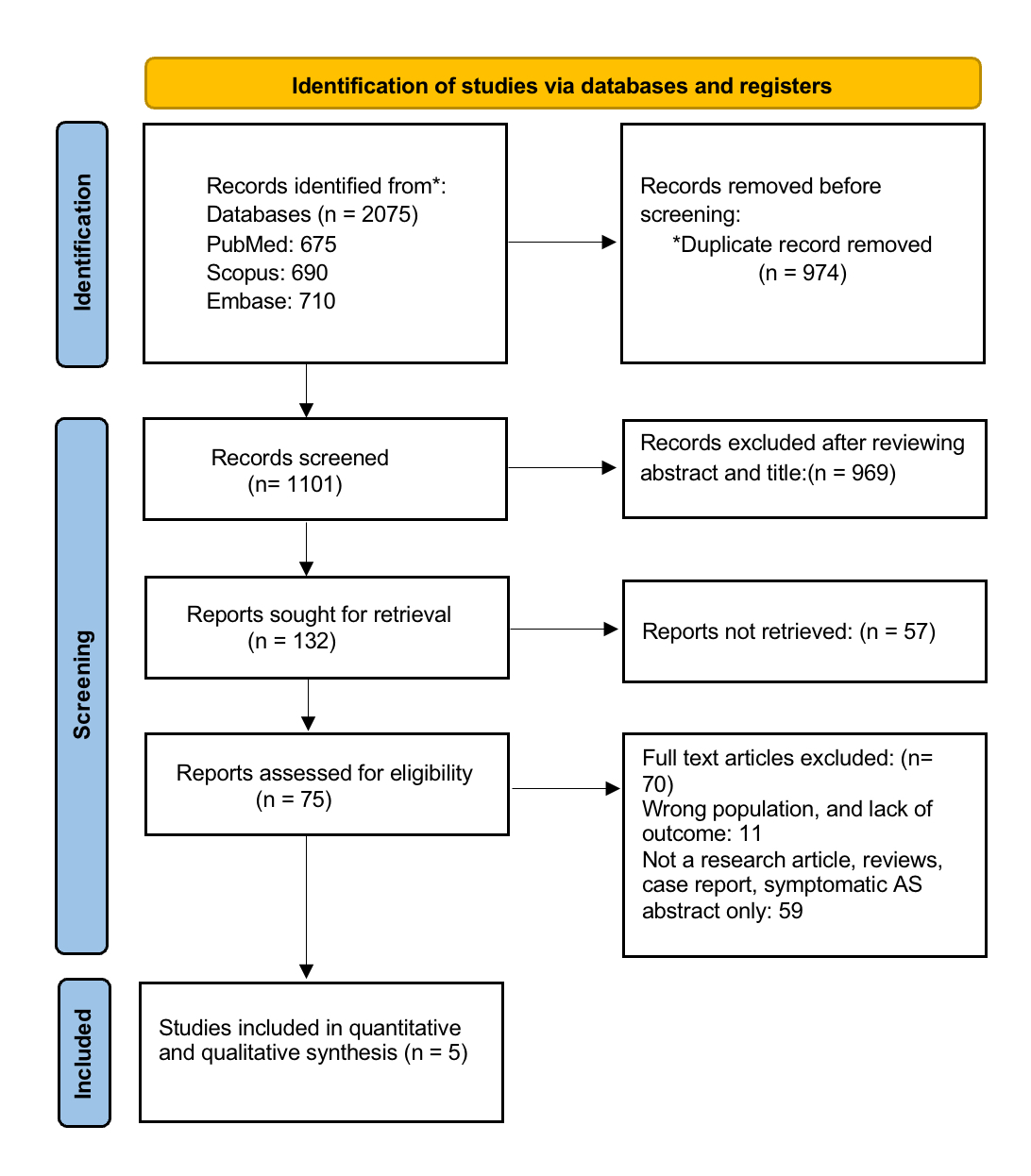


**Supplementary Table 1:** Search strategy

|  | **Search strategy** |
| --- | --- |
| **Electronic database** | (("Aortic Stenosis"[MeSH Terms] OR ("Aortic valve"[All Fields] AND "Conservative management"[MeSH Terms] OR "Watchful waiting"[All Fields])) OR "mortality"[MeSH Terms] OR "cardiovascular outcomes"[All Fields]) OR "cardiovascular mortality"[MeSH Terms] OR "stroke"[MeSH Terms] OR "myocardial infarction"[MeSH Terms] |

**Supplementary Table 2**. Newcastle-Ottawa scale for quality assessment and bias assessment of observational studies.

| Study | Selection | Comparability | Outcome | Total | Quality of Study |
| --- | --- | --- | --- | --- | --- |
| Taniguchi et al, 2015 | **** | * | *** | 9 | High |
| Kim et al, 2019 | **** | * | *** | 8 | High |
| Kang et al, 2010 | **** | * | *** | 8 | High |

*Score >6 was considered as an adequate quality study.

**Supplementary Table 3.** Cochrane Collaboration’s tool for assessing risk of bias in randomised controlled trials

| **Trial Name** | Sequence generation | Allocations concealment | Performance Bias | Detection Bias | Attrition Bias | Reporting Bias |
| --- | --- | --- | --- | --- | --- | --- |
| **AVATAR** | LOW | LOW | LOW | LOW | LOW | LOW |
| **RECOVERY** | LOW | LOW | LOW | LOW | LOW | LOW |
